# Simulation of Attacks for Security in Wireless Sensor Network

**DOI:** 10.3390/s16111932

**Published:** 2016-11-18

**Authors:** Alvaro Diaz, Pablo Sanchez

**Affiliations:** Microelectronics Engineering Group, University of Cantabria, 39011 Cantabria, Spain; sanchez@teisa.unican.es

**Keywords:** WSN, attack simulation, power consumption, security analysis

## Abstract

The increasing complexity and low-power constraints of current Wireless Sensor Networks (WSN) require efficient methodologies for network simulation and embedded software performance analysis of nodes. In addition, security is also a very important feature that has to be addressed in most WSNs, since they may work with sensitive data and operate in hostile unattended environments. In this paper, a methodology for security analysis of Wireless Sensor Networks is presented. The methodology allows designing attack-aware embedded software/firmware or attack countermeasures to provide security in WSNs. The proposed methodology includes attacker modeling and attack simulation with performance analysis (node’s software execution time and power consumption estimation). After an analysis of different WSN attack types, an attacker model is proposed. This model defines three different types of attackers that can emulate most WSN attacks. In addition, this paper presents a virtual platform that is able to model the node hardware, embedded software and basic wireless channel features. This virtual simulation analyzes the embedded software behavior and node power consumption while it takes into account the network deployment and topology. Additionally, this simulator integrates the previously mentioned attacker model. Thus, the impact of attacks on power consumption and software behavior/execution-time can be analyzed. This provides developers with essential information about the effects that one or multiple attacks could have on the network, helping them to develop more secure WSN systems. This WSN attack simulator is an essential element of the attack-aware embedded software development methodology that is also introduced in this work.

## 1. Introduction

Smart environments are increasingly being deployed in building, military, health, ecological, industrial, and transportation applications, among others. These environments are normally based on smart devices that are acquiring data from the real world, processing and communicating these data to information processing centers, generating some information-based services and, sometimes, producing some actions in the environment. The information used by smart environments is provided by Wireless Sensor Networks (WSNs), which are normally responsible for monitoring and recording physical or environmental conditions and communicating the collected data to a central location. These WSNs are a group of spatially dispersed-self-powered sensing devices (nodes). Sometimes, the nodes also integrate actuators and/or displays that provide actions/information to the environment. Commonly, this kind of network consists of a large number (from tens to thousands) of low-cost, low-power, resource-constrained multi-functioning sensor nodes, often operating in an unattended, hostile environment, with limited computational and sensing capabilities [1]. Due to the deployment of these systems, WSNs are especially susceptible to a large number of security attacks [2]. During the last years, a common requirement of WSN specifications is security assessment, especially because of attackers that can disturb the network, access or modify information, affect nodes or modify behavior.

For this reason, it is essential to identify the security weaknesses of the WSN at the earliest stages of the design phase. Furthermore, understanding the impact (especially the power consumption impact) of the most typical attacks on a node (or the entire network) helps to prevent possible problematic vulnerabilities. Thus, attack simulation is of great value when developing the node’s embedded software and/or the complete WSN system.

This work presents an approach to simulate WSNs under different attack conditions, allowing the effects of these attacks on each node and in the whole network to be determined. The proposed work enables the most damaging attacks to be determined in order to help implement/design countermeasures to avoid/detect them.

This approach is very effective because it can be used before network deployment, during the hardware/software design/development phase. It allows developers to design more secure systems and introduce countermeasures to avoid the effects of the most critical attacks.

There are a great number of documented attacks against WSNs [2,3,4,5,6,7,8] and the review analyses presented in [3,4,5,6] show up to 20 different WSN attacks. One of the main problems in developing an attack simulator that implements most of these possible attacks is how to model and classify these attacks. In order to solve this problem, this work proposes an attack model that integrates these attacks in only four attack categories depending on their effects. Each category will be represented by specific attackers in this paper thus facilitating their implementation in any WSN simulator. The combination of these attackers can model most of the reported real WSN attacks.

In the area of attack countermeasures in WSNs there are a wide range of existing techniques (e.g., [2,3,9,10]). In [2], there is a classification of the security goals. They are classified as primary and secondary [11]. The primary goals are the standard security goals such as Confidentiality, Integrity, Authentication and Availability. The secondary goals are Data Freshness, Self-Organization, Time Synchronization and Secure Localization. Shahriar Mohammadi et al. [12] analyze approaches for security detection and defensive mechanisms against link layer attacks. Rajkumar et al. [13] describe different types of attacks, security related issues, and their countermeasures. It also presents some techniques that prevent attackers from accessing the wireless medium, including sleeping/hibernating and spread spectrum communication. Several works describe encryption solutions to achieve security goals in WSNs but, as is commented in [14], it is necessary to measure the effectiveness of these solutions in WSNs. In [15], a physical-application layer security method is presented. These techniques could be implemented in the node software or in the network transceiver.

In addition, this paper also shows how some new attack models are integrated into a WSN platform that is introduced in [16]. A brief introduction of the attack model is presented in [17]. In [17], a first version of the virtual simulator with a reduced group of attacks is presented. There is another work [18] that is focused on the high-level design of the WSN and the attacks with UML-Marte (Unified Modeling Language) models. The WSN simulator executes exactly the same embedded software codes as the physical nodes execute during in-field operation. Therefore, the simulator can generate network traffic that is similar to the real network traffic. This is a very important feature, because the main effect of the attacks is the disturbance of the traffic network. The simulator also has a model of the wireless channel that can be used to simulate any kind of network configuration and deployment. The tool evaluates the behavior of a WSN, taking into account the node’s hardware, the node’s embedded software and network deployment. At the same time, it estimates the node’s power consumption, execution times and security transmissions. An important improvement of this paper is that this framework will be also able to perform WSN attack simulation that enables secure WSNs to be designed and developed. The simulator can obtain execution time and power consumption estimations from any kind of WSN network under attack. Additionally, it provides information about the security encryption used in WSN transmissions. Thus, it is possible to identify the most dangerous attacks and vulnerable parts of the network, prior to any deployment. This is essential to improve Wireless Sensor Network security. Specifically, the main contributions of this paper are:

- A methodology that allows the design of secure WSNs from the first steps of the development process. This methodology uses a WSN simulator to detect potential harmful attacks in a WSN. Moreover, using the simulator it is possible to guide the development and to verify different attack countermeasures.
- A WSN attack model that comprises a large number of attacks in only four categories, thus facilitating its implementation and analysis. This attack model is integrated in a WSN simulator framework that takes into account the network deployment, the Hardware/Software (HW/SW) platform of each node and the actual application code that is executed in each processor. Moreover, the simulator allows the estimation of a metric called “SEM” (Security Estimation Metric) [14] that evaluates the security of the transmissions.
- An attack simulator framework that is able to model, simulate and estimate the impact of attacks over different kinds of networks. It evaluates the node’s software behavior under attack and enables the development of attack-aware embedded software.

The paper has been organized into seven sections. Section 1 presents the paper’s objectives and main contributions. Section 2 reviews previous work related with WSN vulnerabilities and WSN simulators. Section 3 presents the design methodology for secure WSNs. Section 4 explains the WSN simulator. Section 5 describes the WSN vulnerabilities and introduces an attack classification. This section also defines the proposed attackers. Section 6 reports some experimental results. Finally, Section 7 states the conclusions.

## 2. State of the Art

Nodes in Wireless Sensor Networks are usually highly energy-constrained and are often expected to operate for long periods with limited energy reserves. For this reason, early performance estimation is an essential step in any embedded system design methodology. Early, fast and accurate simulations can provide information to the WSN developers that enable the modification of the SW algorithms or the network architecture in order to optimize the WSN design for the best use of the limited resources.

There are several simulators in the literature. In [19,20,21,22], authors present surveys of WSNs simulators. Some of the best known network simulators are NS-2 (The Network Simulator) [23], NS-3 [24], Cooja [25], Castalia [26], OMNET++ [27], GloMoSim [28], TOSSIM [29] and Avrora [30]. NS-2, NS-3 and OMNET++ are discrete event network simulators. NS-2 provides support for simulation of Transmission Control Protocol (TCP), routing, and multicast protocols over wired and wireless networks. There is a NS-2-based open source framework for underwater sensor network simulation called SUNSET [31]. OMNET++ can be used for testing distributed algorithms and/or protocols in realistic wireless channels and radio models, and node behavior, especially relating to radio access. GloMoSim (Global Mobile Information System Simulator) is a scalable simulation library for wireless network systems. It is built on top of the PARSEC simulation environment [32]. Another option, TOSSIM, is a bit-level discrete event simulator and emulator of TinyOS [33] sensor networks. Avrora is a simulation tool which helps to develop sensor network simulation with clock cycle accurate execution of microcontroller programs. Avrora can only emulate two specific platforms. Peng Lei et al. [34] present an extension of OMNET++ framework that provides development interface which uses a UML-RT (Unified Modeling Language extension for Real Time) based model. This framework does not bridge the gaps of OMNET++ (for example, it does not have real Software (SW) code execution and Real Time Operating System (RTOS) support). In [35], UML was applied to model a specific system, consisting in measuring, pre-processing, wireless transmitting and post-processing data from sensors. Luca Berardinelli et al. [36] present an approach for modeling and analyzing the performance of embedded WSN software that is programmed on NesC (component-based event-driven programming language used to build applications for the TinyOS platform). Another simulator is J-Sim [37] a component-based simulation environment developed in Java. The main limitation of this simulator is its low efficiency. UWSim [38] is a simulator used for Underwater Sensor Networks (UWSN). The main limitation of this simulator is that it only allows the simulation of underwater networks. The approach in Shawn [39] is an open-source discrete event simulator designed to simulate large-scale networks from an abstract point of view. However, it does not support real traffic simulation and real SW code. Other simulators include Prowler [40] and JProwler [41]. They are probabilistic wireless sensor network simulators. Prowler is written in Matlab, while JProwler is written in Java. NetTOPO [42] is an integrated framework of simulation and visualization for WSNs. Another approach is ATEMU, [43], a simulator for sensor networks in which the nodes are Crossbow AVR/Mica2 nodes.

Traditional network simulation environments do not capture the operation of endpoint nodes in detail. In most cases, the simulation does not consider the hardware and the software in each node. If this information is ignored, it is impossible to estimate the consumption of the network with precision. Considering that increase in power consumption is one of the main problems of an attacked network, it is very important, in order to obtain valid and accurate estimations, to use a simulator that fulfills some requirements:

- **Use of Real Network Traffic:** In order to estimate the attack effects, it is important to work with the real traffic that the network will have when it is deployed.
- **Simulation of the Real SW Code:** To calculate the behavior of the SW code against attacks, it is very important to simulate the WSN using the final SW code of each node.
- **HW platform support:** The power consumption will vary depending on the Hardware components of the platform. The simulator must support different architectures.
- **OS support:** Nowadays, the software of an embedded system can run over an operating system. It is of great value for the simulator to support different and typical OSs.
- **Power consumption:** The estimation of power consumption is one of the main requirements to estimate the attack effects.
- **Encryption Security Metric:** In order to detect potential vulnerabilities in wireless transmissions, it is important to measure the security of the encrypted packets.

Table 1 presents some simulators considering these requirements. The main lack of these simulators is that none of them has a realistic level of simulation with different operating systems and with power consumption estimation. Power consumption estimation is one of the critical points in these systems. Few of these simulation tools have considered power consumption. They have problems with the scalability of their hardware because they are based on a specific processor or on specific hardware. Another problem with these simulation environments is that they do not simulate the specific software deployed on them, and the network traffic is based on external functions, which is not real traffic. As can be observed in Table 1, the traffic generations of the simulators, in most of cases, are not based on the real traffic generated by the real applications. Normally, the traffic is generated by traffic patterns (for example, in NS simulators, the traffic can be generated with different distributions, such as Pareto or exponential), Statically or Dynamically (for example TOSSIM is a discrete event simulator which can be generated dynamically with external scripts or statically by default) or probabilistic (such as Prowler, which generates its traffic with external functions). Our commitment is to simulate the system performance under attack situations. Without real traffic information it is very difficult, if not impossible, to perform an accurate simulation of real attacks, since their impact on the system behavior mostly depends on corner cases resulting from exploiting the weakness of the application SW. Moreover, none of these simulators provide a metric to measure the security of the encrypted transmissions.

Table 1 shows the main characteristics of the simulator developed where the attack’s model is inserted. As can be observed, this simulator complies with the previous requirements. It has SW and HW support, estimates power consumption and the traffic of the network is generated directly with the software code of each node. Moreover, it supports different Operating Systems such as FreeRTOS, POSIX or WIN32.

There is little work about attack simulation in WSNs. An attack simulator based on the OMNET++ platform and Castalia (ASF, Attack Simulation Framework) is presented in [44,45] but it does not take into account the embedded software. The network traffic is based on external functions in [44,45]. Another framework for the simulation of communication networks attacks is NETA [46]. NETA is built using OMNET++. The problem of this approach is that it only allows the simulation of three different attacks. Analytical models aimed at detecting and contrasting attacks are discussed in [47,48,49]. Y. Xu et al. [49] describe a distributed wormhole detection algorithm, and show simulation results in order to prove its low false tolerance and false detection rates. S. Kaplantzis et al. [50] propose an intrusion detection scheme which detects black hole and selective forwarding attacks. In these cases, simulation is used to validate their correctness and efficiency. Y.-T. Wang et al. [51] present a framework that simulates occurrence of attacks by injecting events into real application simulators. In [52], UML Sequence Diagrams are used to describe and analyze possible attacks in a network and transport layer.

Some of the previously commented simulators can provide WSN simulation with HW/SW support, RTOS support and power/execution time estimation but, as far as the authors know, there is no simulation framework that integrates all these features and/or attack simulation.

## 3. Secure WSN Design Methodology

**Figure 1:**
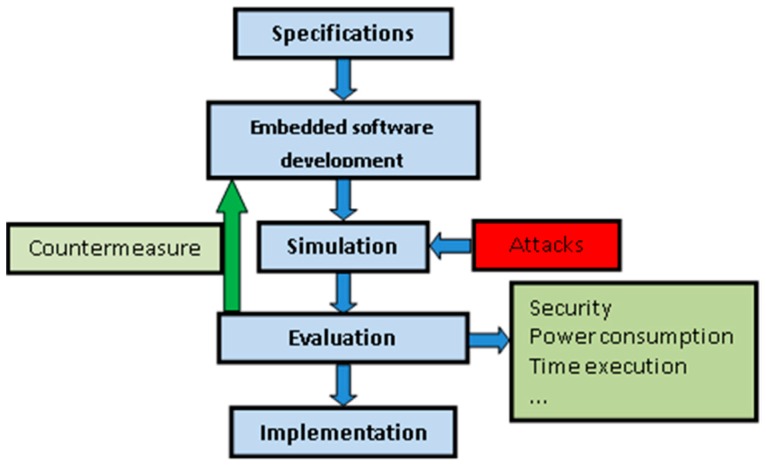
Firmware-aware attack design.

This paper presents a methodology to design attack-aware embedded software/firmware or attack countermeasures. It makes use of a WSN simulator that includes attack simulation. The proposed methodology is presented in Figure 1. It includes a simulator that allows the evaluation of the network behavior under different conditions (different network topologies, attacks, software versions, etc.). The estimations that the simulator provides allow the detection of the most harmful attacks and the most vulnerable nodes or configurations. These evaluations of security, power and behavior also help developers to select which countermeasure is added. The data obtained from the simulation can be used by developers to evaluate the attack effects on the network nodes. As a result, developers can design, develop or modify a custom countermeasure for each network node. Once a countermeasure is implemented, it can be tested with a new simulation. As shown in Figure 1, in the evaluation step of the methodology, developers can explore and compare the effects of the attacks with different configurations or countermeasures. This enables the modification of the application software or hardware of the nodes in order to improve the performance of the network. Additionally, this methodology allows the comparison of different countermeasures, thus only the most efficient are implemented in the final systems. These countermeasures can be selected from a wide range of existing techniques in the state-of-the-art or can be designed with the support of the simulator.

The countermeasures and/or attack-aware embedded software are evaluated in normal conditions and under attack with the proposed methodology. Different types of attacks are injected in order to evaluate the behavior and performances (mainly power consumption, response time and active period) of the WSN nodes. The simulation also takes into account the network deployment.

The proposed methodology includes two main stages:

1. **Evaluation of attacks:** This stage evaluates the impact of attacks in each network channel and/or node. The effect of an attack is different depending of the topology of the network, node software, HW components or even the node/network configuration. An attack can be very harmful for a specific node but harmless to another node. Thus, the WSN simulation will help to identify the most problematic attacks and which parts of the WSN could be most compromised. With the proposed virtual platform, it is possible to simulate a sufficiently accurate hardware and network model under attack conditions while the real embedded software is being executed in the nodes. With the simulation and performance results, it is possible to identify the most dangerous attacks for the WSN.
2. **Select a countermeasure:** The previous stage enables the detection of the most harmful attacks, thus the next step is to select and evaluate the possible countermeasures. Additionally, it is also possible to use the estimations to modify the embedded software and minimize the attack effect. For this reason, this stage includes two steps:
  - **Design of an attack detection procedure:** Once the most dangerous attacks are identified, it is necessary to develop firmware/software that detects when the system is attacked. In order to guide this process, the evaluation of the system behavior and estimations provided by the WSN simulator are studied to find the effects that the attacks produce. The objective is to identify a method to detect when a node is being attacked so that a solution to that attack can be implemented. For example, if the network is simulated in normal conditions (without attacks) a rate for the transmitted and received packets can be obtained for a particular network deployment. If the same network is simulated with the injection of a jamming or replication attack, the packet rate in the network and/or in some nodes could change. With the estimations that the virtual simulator provides, the developers can use the attack effect to detect the instant in which an attack takes place. In the case of a jamming attack, the traffic rate varies compared with normal conditions. Because of this variation, it is possible to define a range for normal traffic in a particular deployment. Thus, when this range is violated, the node could assume that it is under attack.
  - **Design of attack countermeasures:** Once an attack is detected, a countermeasure must be executed. These countermeasures should have minimum effect in the normal behavior of the network. Moreover, they should avoid the effects that the attacks produce. With these objectives, the software developers can design the countermeasures and test them in the virtual platform, before network deployment. These attack countermeasures may use different techniques. The most common methods include turning off attacked nodes, changing the wireless communication channel, changing the encryption key of the communication messages or even excluding the attacker from the network using a filter. The countermeasures are not limited to these methods but they can be as sophisticated as the developer or application requires. The advantage of the proposed methodology is that these countermeasures can be evaluated and improved before network deployment. Thus, faults and inefficient implementations can be detected in the early stages of the design process and fixed at low cost. In addition, this methodology allows the comparison of different countermeasures, thus only the most efficient is implemented.

## 4. Secure WSN Design Methodology

The objective of the framework is to enable the simulation of WSNs in order to analyze the effects of different attacks on the system. The simulator allows designers to evaluate the network/node behavior in an easy-to-use loop where different design alternatives can be evaluated in the first steps of the design process. The simulator will use the same source code that is executed in the real network nodes.

**Figure 2:**
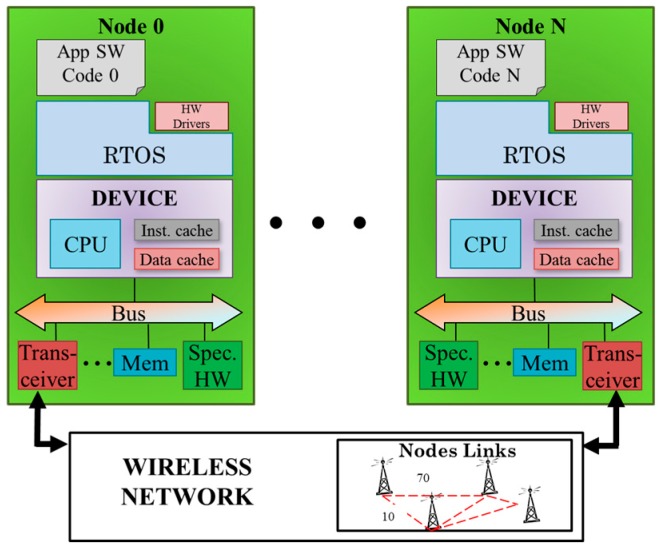
Scheme of Wireless Sensor Network virtual platform without attack model.

The WSN virtual platform presented in this paper is based on the native-simulation approach. In this approach, the source code of the WSN nodes is instrumented with additional code that model target-platform (WSN mode) performance or specific characteristics. The instrumented code is executed in a host platform (desktop computer) and it provides estimations during execution. As is shown in Table 1, the simulator has some novel features that improve simulation accuracy and facilitate attack simulation. The virtual platform is a software model of the WSN that enables system simulation. It includes models of the main elements of a WSN: network model and node model. The node model integrates processors, memories, RF-transceivers and sensors (see Figure 2). This allows the analysis of the functionality of each WSN node and the estimation its temporal and power consumption behaviors. These estimations are relevant features in order to evaluate the damage/impact of attacks. It also has a reliable network model than can be modified to evaluate any kind of network topology and deployment.

### 4.1. Simulation Technique

**Figure 3:**
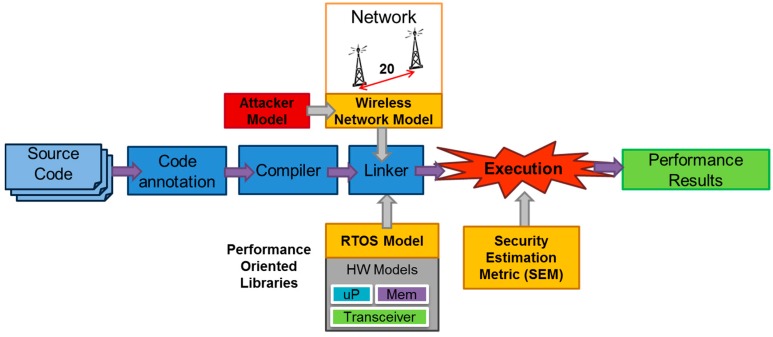
Native/Host-compiled co-Simulation process in the WSN virtual platform.

The simulation methodology used in this work is based on the native simulation approach [53] depicted in Figure 3. The simulator presented in [53,54] (SCoPE) extends SystemC capabilities to perform HW/SW co-simulation. SCoPE is used as a starting point of this work that extends this simulator with new capabilities such as WSN and attack simulation. The simulator supports platform modeling for behavioral simulation and performance estimation of embedded systems. The native-simulation based frameworks model all hardware elements related with software execution (e.g., processors, memories and buses) and RTOS with a set of tables that define the execution time and power consumption per instruction and RTOS function [52]. This approach combines the execution of the annotated software code in a host platform with the use of a virtual platform model of the hardware architecture and embedded RTOS. With this simulation technique, it is possible to model hardware platform components in System-C and execute the software code of each node on the same platform. D. Calvo et al. [54] present several high-level component models (e.g., processors) and how the power estimation is implemented. In Section 4.2, this work defines new models that are essential components of a wireless sensor node. Thus, it is possible to obtain fast and accurate estimations of power consumption, execution time, data/instruction cache hits and misses, number of bus accesses, network traffic, etc. The co-simulation process includes several steps:

- The embedded source code is parsed and analyzed. The basic blocks are identified and annotated with several performance-oriented parameters (energy consumption and execution time per basic block, cache and bus access requirements, etc.).
- The instrumented code is compiled and linked in a host computer (host-compiled or native simulation) with several additional libraries that implement platform HW/SW components or network models: processors, network, RTOS, bus, cache, etc. This compilation process generates an instrumented executable to simulate the system. The execution of this code will produce the performance analysis results.

The framework also includes RTOS (Real Time Operating System), network and attacker models (see Figure 3). Additionally, the simulator calculates a Security Estimation Metric during simulation. This metric provides information about the robustness of the encryption that has been used in the WSN transmissions. This metric is presented in [14]. The RTOS library was presented in [16] and it provides functional and temporal behavior to the RTOS API (Application Programming Interface) functions. Thus, the node’s software with RTOS function calls that is executed in the real nodes can be directly executed in the virtual platform. The network model and the attacker model libraries will be presented in the next subsections.

### 4.2. WSN Specific Hardware Components

There are two essential components in a wireless sensor node that other systems normally do not integrate. These components are the sensor and the RF transceiver. The sensor is responsible for collecting external information with a certain period or when an event occurs. This is implemented in the simulation as an external component with specific power consumption and response time. The sensor model mainly includes the information that has to be transferred to the node, its power consumption, response time and active period. Another important component is the RF transceiver. This is more complex than a sensor and typically integrates a configuration register to control its operation mode. This paper includes a use-case in which the RF transceiver models an 802.15.4 XBee radio from Digi International [55]. In this case, the implemented registers were:

- **Destination Address High and Low:** These registers define the message destination address.
- **Baud Rate:** Speed for data transfer between transceiver and WSN node controller.
- **Mac Retries:** Number of retries that can be sent.
- **Multiple Transmissions:** Number of additional broadcast retransmissions.
- **Power Level:** RF module transmission power.

### 4.3. Wireless Sensor Network Model

In wireless transmission, the physical channel between two nodes is a shared channel, with limited range, noise and interference. Additionally, the messages can be listened to by other nodes that are not the destinations of the packet. As a consequence, developers need to determine the node visibility and the probability of a non-successful reception of a packet (packet loss probability). The WSN deployment area has to be analyzed and a matrix with the probability of packet loss among all nodes has to be defined. The matrix of packet-loss probability models RF channel characteristics. This probability data may be calculated by the user or by external tools such as an electromagnetic-propagation simulation tool such as Cindoor [56]. With this matrix, the virtual platform can estimate the connections of the network and the effectiveness of the links between nodes.

**Figure 4:**
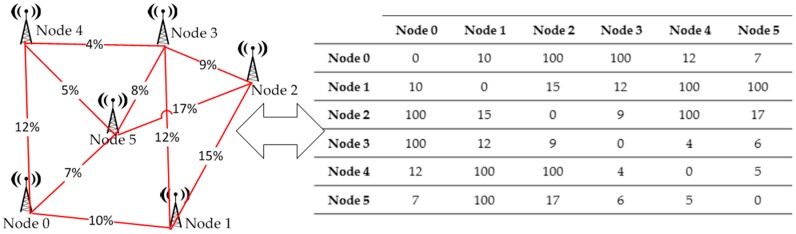
Wireless Network Model and packet-loss probability matrix.

For example, a 100% packet-loss probability means that the sending-node range is not enough to directly reach the destination node, thus all the transmitted packets are lost. If the link has a 0% packet-loss probability, all the packets reach their destinations. If the link from node 0 to node 1 has a probability of 10% (Figure 4), this indicates that, on average, 90 packets will reach node 1 for each 100 packets transmitted by node 0.

**Figure 5:**
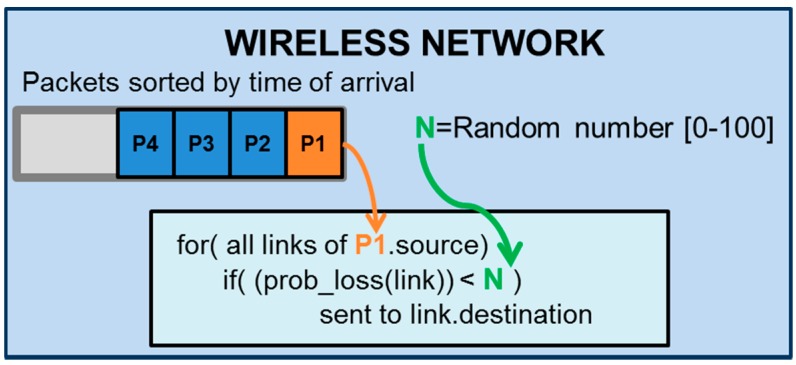
Normal network mode operation.

It is important to clarify that the network model is responsible for transmitting the packets to their destinations. When a node sends a packet, the network adds the packet to the transmission queue that is sorted be the time of arrival at the reception node. When the simulation time matches the time of arrival of the packet, the wireless network pops the packet and generates a real random number between 0 and 100. If the packet-loss probability (“prob_loss (link)” in Figure 5) is lower than this random number, the network model transmits the packet to the destination node; otherwise, the packet is discarded. Figure 5 represents a scheme of this wireless network operation.

In the network simulator, another important element is the node network interface. This interface is responsible for deciding which packets should actually be received by the node. In a real wireless network, when a node sends a packet to another node, this packet is not only received by the receptor node but also by all the nodes in the transmission range of the sender. In this case, the node network interface is responsible for disposing of the packets that do not correspond to the node. The network interface checks the packets and the transmission times. In case of package collision (two or more packets are overlapped on time), the network interface will discard all the packets involved in the collision. The interface could also implement the network protocol (Zigbee, 802.11, 802.15.4, etc.). This allows the modeling of real network transceivers that integrate RF modules with a microcontroller for network protocol management (for example, the Xbee [55]). The current version of the network interface implements the Zigbee protocol with a C/C++ code. Different protocols and networks interfaces can be implemented with a modification of the C/C++ module code. This facilitates the evaluation of different network protocols.

## 5. Modeling of WSN Attacks

The previous section presents the infrastructure that allows the simulation and exploration of different WSN design alternatives in normal operation. However, as was mentioned previously, in many cases WSNs are deployed in hostile environments that could put at risk the system behavior. Thus, during the WSN specification and design process, designers must take into account a wide range of unexpected events (attacks) that can impact the WSN’s expected behavior.

### 5.1. WSN Attacks

In order to improve network security, it is important to identify the most harmful attacks that a network can suffer. An attack can be defined as an attempt to gain unauthorized access to a service, resource or information. It could also be an attempt to compromise integrity, availability, or confidentiality of a system. The nature of the attacks is huge enough to make them difficult to classify. Nevertheless, Mohammadi, S. et al. [57] distinguished two large attack categories: passive and active attacks.

While passive attacks relate to privacy (eavesdropping, gathering and stealing of information by intercepting data communications or monitoring packets exchanged within a WSN) active attacks perform actions such as injecting faulty data into the WSN, impersonating, modifying resource and data streams, creating holes in security protocols, destroying sensor nodes, degrading performance, disrupting functionality and overloading the network.

The model and tool presented in this paper are focused on active attacks, which mostly affect network performance. More precisely, this paper addresses those attacks that disrupt, totally or partially, the communication flow among network nodes. Typical WSN attacks can be classified into different categories, according to [3].

A detailed study of these attacks concludes that they mainly produce two effects. The first effect is the increase in the network traffic due to the introduction of new packets in the network. The second one is the opposite effect, the reduction of the network traffic due to the elimination or loss of network packets. These two effects may act together increasing the network traffic of a specific packet and decreasing others. A more detailed study of the attacks shows that not all the attacks can be modeled with these effects, thus an additional special model is required.

In summary, WSNs attacks can be classified in four categories depending on their effects on the network:

- Attacks that introduce packets in the network.
- Attacks that introduce noise in the network.
- Attacks that introduce noise and packets in the network.
- Attacks that modified the firmware of a node.

This section presents a model that groups most of the WSN attacks in four categories, in terms of the attack effects on the network.

#### 5.1.1. Attacks That Introduce Packets in the Network

The first type of attacks are based on the introduction of fake packets into the network with the aim of making the original nodes process them, increasing the traffic in the network and, thus, congesting it or even disrupting the data of the network.

- **Interrogation attack:** This attack exploits the two-way RTS/CTS (Request to Send/Clear to Send) handshake that many media access control (MAC) protocols use to mitigate the hidden-node problem. The attacker repeatedly sends RTS messages to obtain CTS responses from a targeted neighboring node.
- **Energy Drain [58]:** Due to the difficulty of replacing sensor node batteries and their energy constraints, attackers may use compromised nodes to inject fabricated reports into the network or generate large amounts of traffic in the network. These fake messages cause false alarms that waste response effort, and drain the finite amount of energy in a battery-powered network. The aim of this attack is to destroy the sensor nodes in the network, degrade performance of the network and eventually split the network grid up, so taking control of part of the sensor network by inserting a new Sink node.
- **Hello Flood attack [59]:** The attacker typically attempts to drain the energy from a node or exhaust its resources. An attacker with large transmission power could broadcast “HELLO” packets (used in many protocols for node discovery) to convince every node in the network that the adversary is within one-hop communication range, causing a large number of nodes to waste energy sending packets to this imaginary neighbor and thus into oblivion.
- **Misdirection attack [60]:** The attacker routes the packet from its children to other distant nodes, but not necessarily to its legitimate parent. The main objective of the intruder is to misdirect the incoming messages to increase the latency, which prevents a few packets from reaching the base station.
- **Flooding attack [61]:** An attacker may repeatedly make new connection requests until the resources required by each connection are exhausted or a maximum limit is reached. It produces severe resource constraints for legitimate nodes.

#### 5.1.2. Attacks That Introduce Noise in the Network

The effects of the attacks placed in this group consist in reducing the traffic in the network. These attacks are focused on the introduction of noise in the network (or other techniques) with the objective of increasing the probabilities of packet loss. The main consequence of these attacks is the increment in the packet loss rate which can disrupt the proper function of the network. The attacks placed in this category are the following:

- **Jamming attack [62]:** This works by denying service to authorized users as legitimate traffic is jammed by the overwhelming amount of illegitimate traffic. It disrupts network functionality by broadcasting high-energy signals. There are many Jamming attack strategies.
- **Collision attack [63]:** In a collision attack, an attacker node does not follow the medium access control protocol and produces collisions with the neighboring node’s transmissions by sending a short noisy packet. Packets collide when two nodes attempt to transmit on the same frequency simultaneously, producing packet corruption. This attack can cause a lot of disruption to network operation.
- **Resource Exhaustion attack:** Operation of this attack consists in repeated collisions and multiple retransmissions until the node dies. A malicious node continuously requests or transmits over the channel.
- **Black Hole attack [64]:** A black hole attack basically consists in the network routing alteration with the objective of attracting all the packets to the attacked node destination, and silently discarding or dropping them.
- **Denial of service (DoS) attacks [65]:** In a Path-based DoS (PDoS) attack, an adversary swamps sensor nodes a long distance away by flooding a multihop end-to-end communication path with either replicated packets or spurious injected packets. It can cause serious damage in resource-constrained systems.
- **Homing attack:** In a homing attack, the attacker looks at network traffic to deduce the geographic location of critical nodes, such as cluster heads or neighbors of the base station. The attacker can then physically disable these nodes. This leads to another type of black hole attack.
- **Selective Forwarding attack [66]:** Multi-hop networks assume that participating nodes will faithfully forward and receive messages. However a malicious node may refuse to forward certain messages and simply drop them, ensuring that they are not propagated any further. The procedure to launch selective forward attacks is very similar to the black hole one. First, a malicious node has to convince the network that it is the nearest node to the base station, attracting network traffic to route data through it. Then, a selection of packets is dropped.

#### 5.1.3. Attacks That Introduce Noise and Packets in the Network

The third group consists in attacks that cause different effects on the network by mixing effects of the previous ones. These attacks cause the network to lose some packets, but simultaneously they introduce new packets in the network. Thus, these attacks alter the types of packets transmitted, by reducing the number of some types of packets but increasing others, with the consequent impact on the WSN.

- **Spoofed attack [67]:** A spoofing attack is a situation in which an attacker successfully masquerades as another node by falsifying data and thereby gaining an illegitimate advantage. This attack consists in targeting routing information while it is being exchanged: creating routing loops, attracting or repelling network traffic from selected nodes, extending and shortening source routes, generating fake error messages, partitioning the network, etc.
- **Sybil attack [68]:** This attack consists of the modification of the network routing to attract the traffic of the attacker nodes, with the objective of isolating these nodes. When these nodes can no longer communicate, the attacker sends fake traffic supplanting the nodes.
- **Node replication attack [69,70,71]:** This is an attack where the attacker tries to mount several nodes with the same identity at different places in the existing network. Although Sybil attacks and Node Replication attacks might seem similar, these attacks are essentially different. In Sybil attacks, a single node exists with multiple identities while in node replication attacks multiple nodes are present with the same identity. Therefore, in Sybil attack an adversary can succeed by mounting only a single node, whereas a node replication attack requires more nodes to be mounted throughout the network. In this way, as the number of network nodes increases, the chance to detect this attack also grows.
- **Looping in the network attack:** This attack consists of the modification of the network routing by affecting the node data transmission. A “fake” node informs another node that it is the final node in the chain or that it is the closest node to the end of the node chain. This “fake” node re-sends the packet again to the network, enclosing the packet in an infinite loop.

#### 5.1.4. Attacks That Modify the Firmware of a Node

Finally, some attacks do not directly affect the network traffic but they could affect the software/firmware or the hardware of the node. These attacks usually require direct access to the hardware node (tamper attacks).

- **Application attack:** This attack modifies the firmware/software that is stored in a node. It normally requires access to the on-field software update procedure (Over the Air Programming procedure) or physical access to the hardware of the node.
- **Overwhelm attack:** An attacker might attempt to overwhelm sensor nodes with sensor stimuli that could produce large volumes of traffic to a base station. This induces, among other problems, a power consumption increase in the attacked nodes and the generation of unreliable sensor info.

### 5.2. Attackers

This section defines three attackers that will cover all the attacks presented in the previous subsection. As is mentioned previously, the studied attacks have four different effects. These effects can be modeled with only three attackers. Therefore, all the mentioned attacks can be modeled with a combination of these three attackers.

#### 5.2.1. Increased Network Traffic

A typical attack could increase or reduce the network traffic. For example, the attacker repeatedly sends RTS (Request to Send) packets into the network in the “Interrogation attack”. In a “Hello Flood attack”, the attacker typically attempts to drain energy from a node injecting “HELLO” packets. It can be observed that both attacks are similar: the attacker injects different types of packets in the node. Thus, this type of attacks could be modeled with the same attacker node: a “**Fake packet injector Attacker**”. This special WSN node is responsible of introducing fake packets in the network. Depending on the real WSN attack that is been analyzed, this attacker node injects a different kind of packet (“Hello”, “RTS”, data, etc.) into the network. The structure of this package can be user-defined. The packets are received by the WSN nodes because their structure is formally correct. The simulation of this kind of attackers depends on several parameters that have to be defined during attack configuration. The “Fake packet injector” attacker requires several parameters:

- **Frequency:** It defines the fake packet rate or number of packets per second that are injected into the network.
- **TypePackets:** It defines the type of packets that the attacker injects. Several types of packets have been implemented: “HELLO”, RTS, CTS and random payload packets. The structure of this package can be user-defined.
- **Time:** It defines the range of time in which the attacker is active. The attacker can be turned on and turned off many times during the simulation.
- **Nodes Destine:** This list specifies the WSN nodes that receive of the injected packets.
- **Broadcast:** If this attribute is defined, each packet will be sent to all nodes.

In summary, the attacks that have the effect of increasing the network traffic could be modeled with a “Fake packet injector” node (attacker) with specific parameter values.

#### 5.2.2. Reduction of Network Traffic

Other attacks could reduce the network traffic. They could be modeled with a new type of attacker: the “**Link Noise Attacker**”. For example, in a “Jamming attack”, the attacker disrupts network functionality by broadcasting high-energy signals. The packets that are affected by these high-energy signals cannot reach their destination (the destination node only receives noise and it is not able to decode the packet). In the case of a black hole attack, the network routing structure is modified with the objective of attracting all the packets to the attacker node. Then the attacker silently discards or drops all the captured packed. As can be appreciated, the effects of both attacks are similar: there is a reduction of the communication link quality in the network. Therefore, it is possible to use the same attacker to model both attacks, a “Link Noise attacker”. This attacker is responsible for introducing noise in the network with the objective of increasing the probability of packet loss.

This paper presents a WSN simulator with a wireless channel model that takes into account the WSN deployment. This model associates packet-loss probabilities to every possible wireless channel between nodes. This feature is used to define the “Link-Noise attacker”.

A “Link-Noise attacker” dynamically modifies the packet-loss probability that is associated with every pair of nodes in the WSN simulator. This reduces the communication link quality, thus packets could fail to reach their destination. In order to define a Link-Noise attacker, several parameters can be specified:

- **Links:** List of communication links or node-pairs that are affected by this attacker node.
- **Power:** Noise that will be applied to every link that has been defined in the previous parameter. It specifies the additional percentage of packet-loss probability that will be added to the original link’s packet-loss probability.
- **NumPackets:** Percentage of packets that will be affected by the increased packet-loss probability. For other packets, the packet-loss probability will not be affected.
- **Time:** It defines the range of time in which the attacker affects the network. The attacker can be turned on and turned off many times during the simulation.
- **TypePackets**: The attack will only be active for specific packet types. This enables the simulation of selective attacks.

#### 5.2.3. Increase and Reduction of Network Traffic

The “fake packet injector attacker” and “link-noise attacker” are not enough to model all the attacks that were described in the state-of-the-art section. Some attacks could be modeled with a combination of both attackers. For example, a “Sybil attack” can be modeled with a “link-noise attacker” (which isolates a node) and a “fake packet injector” (which injects the fake traffic of the isolated node in the network). Another example of an attack modeled with the combination of both attackers is the node replication attack. It is an attack where the attacker tries to mount several nodes with the same identity at different places in the existing network. To model this attack, the “link-noise” attacker isolates the nodes to be replicated, disabling these nodes from receiving any packet. The second attacker node, the “fake packet injector” is responsible for injecting the packets supplanting the isolated nodes.

#### 5.2.4. Attacks to Node Software and Hardware

A more extended study of the WSN attacks shows that not all the attacks can be modeled with the “fake packet injector” and “link-noise” nodes. This is the reason why a special attacker is proposed: the “Direct attacker”. For example, the “application attack” modifies the firmware (embedded software) that is executed in the attacked node. The “Direct Attacker” modifies the node’s embedded software, thus the attacked node will have different behavior. This attacker will accept two parameters that define the new application code that will be downloaded to the node and the time in which the node will be attacked.

### 5.3. Relation between Real Attacks and Proposed Attacker Models

The previous section defines three new attacker models that emulate most of the real WSN attacks. These attacker models are:

- **“Link-Noise Attacker”:** Inject noise in the channel.
- **“Fake packet injection Attacker”:** Inject packets in the network.
- **“Direct Attacker”:** Direct attack on a node.

Table 2 shows the relation between real WSN attacks and the proposed attacker models. The set of real attacks presented in this section is based on the vulnerabilities described in the state-of-the-art. These real attacks will be modeled with a proposed attacker or a combination of them. The proposed attacker and the real attack must provide the same effect in the WSN. As can be seen in Table 2, a Link-Noise node can model different attacks: Jamming, Collision, Black Hole, Resource exhaustion, Homing Selective Forwarding and Path-based attacks. Different parameter values of the attacker node configuration enable different types of attacks to be modeled. For example jamming and collision attacks can be modeled with the same attacker node (Link-node) but with different parameters. These parameters are not only used to define attacks, but also to define attack strategies.

The Interrogation, Energy drain, Hello Flood, Misdirection and Flooding attacks are modeled by a “Fake Packet injector” attacker.

The combination of the Link-Noise node and the Fake Packet injector node enables the simulation of the Spoofed, network Looping, Sybil and Node Replication attacks.

The Direct attacker enables the modeling of the Overwhelm and Application attacks. The tampering and sniffing attacks are not modeled because they are passive attacks and they do not affect the operation of the node. In this case, the tampering attack is based on giving physical access to a node; an attacker can extract sensitive information such as cryptographic keys or other data on the node.

As can be appreciated, most WSN attacks can be modeled using the proposed attackers. For this, it is necessary to focus on the effects of each attack and use different attackers to simulate each effect.

### 5.4. Attackers in the Virtual Platform

**Figure 6:**
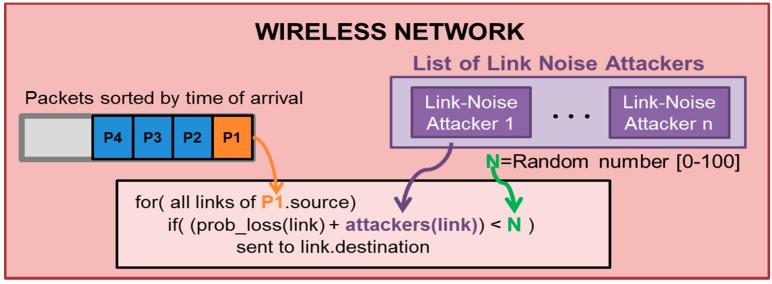
Network mode operation for Link Noise Attackers.

In order to simulate “Link-Noise” attackers, the previously commented WSN network model (Figure 5) has been modified according to this attacker. Basically, this attacker modifies the packet-loss probability for certain packet types during predefined periods of time. The modification is presented in Figure 6. When a packet has to be transferred to the receiver node, the reception probability will include the original link probability and the additional noise produced by the attacker.

**Figure 7:**
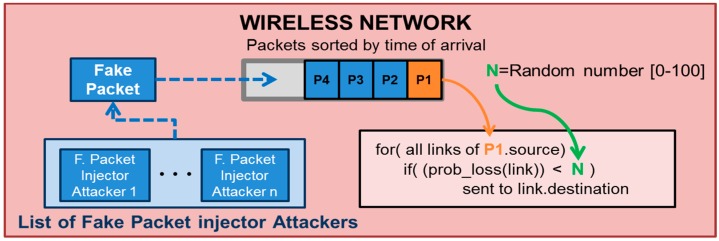
Simulation with Fake Packet Injection attackers.

If a Fake packet injector attacker has to be simulated, new packets have to be injected into the transmission queue of the network model shown in Figure 5. Once the packet is inserted in this queue, the network transmits these fake packets as if they were genuine. Figure 7 shows how this attacker modifies the network model in Figure 5. The “Fake packet attacker” is responsible for generating the fake packets with the structure defined during the attacker configuration and introducing them directly into the transmission queue.

The direct attacker modifies the node’s embedded software. It models the programming of a genuine node by a fake program. The attack definition includes the new application to be downloaded to the node and its network parameters (packet-loss probabilities).

## 6. Experimental Results

In order to evaluate the proposed attack modeling and simulation techniques, two types of experiments have been performed. The first type of experiments (Section 6.1) will evaluate the impact of attacks on different network topologies with the proposed virtual platform. The second type (Section 6.2) will evaluate the accuracy of the proposed simulation technique. This section also demonstrates the advantages of the attack-aware software that was developed with the proposed methodology.

### 6.1. Analysis of the Attack Impact (Experiment 1)

**Figure 8:**
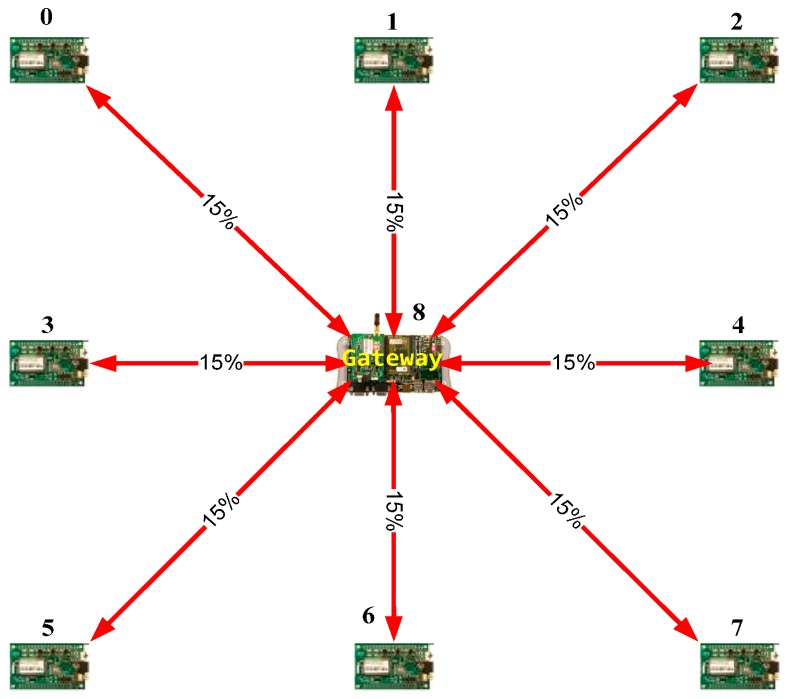
Meshed network example.

**Figure 9:**
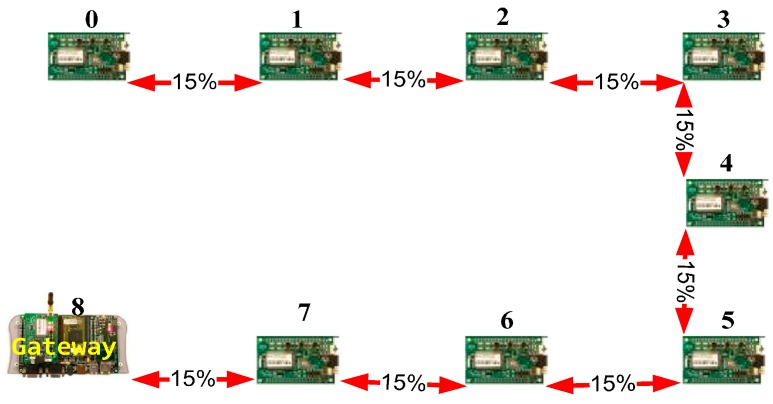
Linear network model example.

**Figure 10:**
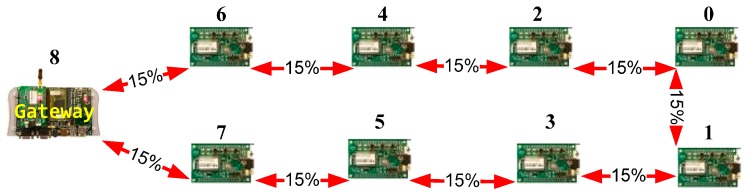
Circular network model example.

In order to demonstrate the suitability of the attack simulation, three network topologies will be evaluated. The three networks have nine nodes with similar hardware architecture and embedded software. These nine-node networks are shown in Figure 8, Figure 9 and Figure 10.

The first one is a meshed wireless network (Figure 8). Figure 9 shows the deployment of a linear wireless network and Figure 10 shows a circular wireless network. The percentages on the red lines represent the packet-loss probabilities of the wireless channel. If there is no red line between two nodes, it will be assumed that the packet-loss probability is 100% (no direct connection). In these examples, the packet loss probability is always the same (15%).

The simulated networks include two different types of nodes: the gateway and the sensor nodes. The Gateway or Central node is responsible for coordinating the network and communicating with other external networks. The Sensor node has a sensor to read the environmental temperature. When the nodes finish their operation, they change to a sleep mode to reduce power consumption and to increase the battery life. All the simulated nodes integrate a Cortex M4 microcontroller, running at 90 MHz, a memory and an 802.15.4 XBee transceiver [55].

Each node type (Gateway and sensor) runs different embedded software with the same RTOS, FreeRTOS. The basic functionality of every node type is briefly commented:

- **Gateway:** The Gateway is responsible for receiving messages from the Sensor nodes and transmitting them to an external network. When all the Sensor Nodes are awake, the Gateway waits for all responses with data about environmental temperature. When the Gateway has the information from all of nodes, it composes a message and sends it through a GPRS module. After this, the node sleeps for about 30 s.
- **Sensor:** After waking up, it reads the sensor and sends the data to the Gateway or to another node that can reach the Gateway node. When it finishes its function, it sleeps for 30 s. Thus, the frequency of data acquisition is every 30 s.

Three different attacks have been analyzed. The first attack is a jamming attack on the gateway nodes with an effectiveness of 60%. The second attack is a Hello Flow (Interrogation) attack on the gateway nodes and the third attack consists in a jamming and an injection attack on node 3. The objective of these simulations is to show how the tool can estimate the attack impact (in terms of energy consumption) for each node and the network.

#### Results of the Attack Simulation

This section presents the virtual simulator results. To obtain these results, each network is simulated during one hour. The energy consumption estimations for each node and for the whole network are shown in Table 3, Table 4 and Table 5. The energy consumption values are shown for the no-attack case. In the case of attack, the tables show the energy increase that the attack produces. It is represented as a percentage: 0% means that there is no energy increase while 100% means that the attacked node requires double the energy.

Table 3 shows the results for the linear network. It can be observed that the injection attack increases the gateway energy consumption by 543%, compared to the no-attack case. However, if the total energy consumption of the network (“TOTAL” column) is taken into account, the “Jamming + Injection attack” at node 3 produces the highest impact. In the case of the linear network, the effect of the Jamming attack is low compared to the other two attacks.

Table 4 shows the results for the Meshed network. It can be observed that the jamming attack has the highest network impact. Specifically, this attack doubles the energy consumption of the network compared to the no-attack case. It affects all the nodes that compose the network almost equally. For this topology, the Injection attack produces the smallest impact.

Table 5 shows the results for the Circular network. It can be observed that the Jamming and Injection attack have a similar impact on the whole network. However, the “Jamming + Injection” attack produces an important impact on this type of network.

**Figure 11:**
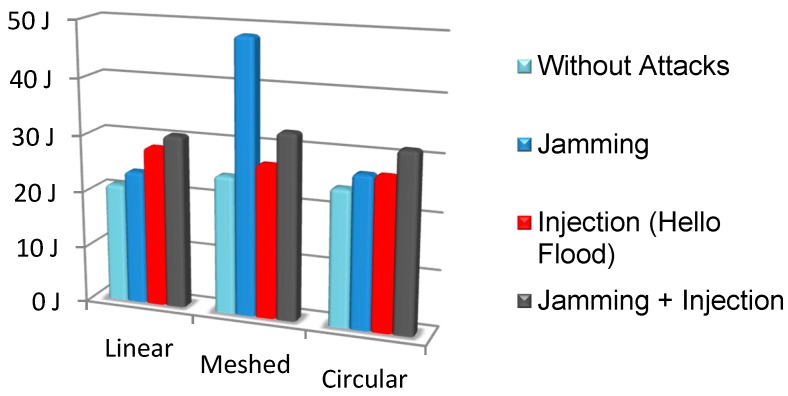
Total Energy consumption.

Figure 11 shows the absolute values of the total energy consumption for the 12 different cases. It can be observed that the Meshed network suffers a great impact under a Jamming attack. Thus, embedded software should integrate some countermeasures to reduce the impact of this type of attack. Additionally, the “Jamming + Injection attack” normally has more impact than other attacks (except for the Meshed network), thus this should be taken into account during embedded software development for linear and circular network topologies.

In addition, it is important to notice that the virtual platform provides estimations that are useful even for no-attack cases. For instance, the total energy consumption of the linear topology (for the specific software and hardware that has been used in the simulated nodes) is lower than for the other topologies. The estimation of the node energy consumption also enables the estimation of the node battery life that is an essential parameter of WSN.

The time of all simulations is similar and it is less than 1 min depending on the traffic network. Table 6 shows simulation times of the experiments included in this section which are performed in core i5-3470 3.20 GHz with 4 Gb RAM in a Fedora 32 bits. This is a very low simulation time when taking into account that the simulated time is 1 h.

### 6.2. Attack-Aware Software (Experiment 2)

**Figure 12:**
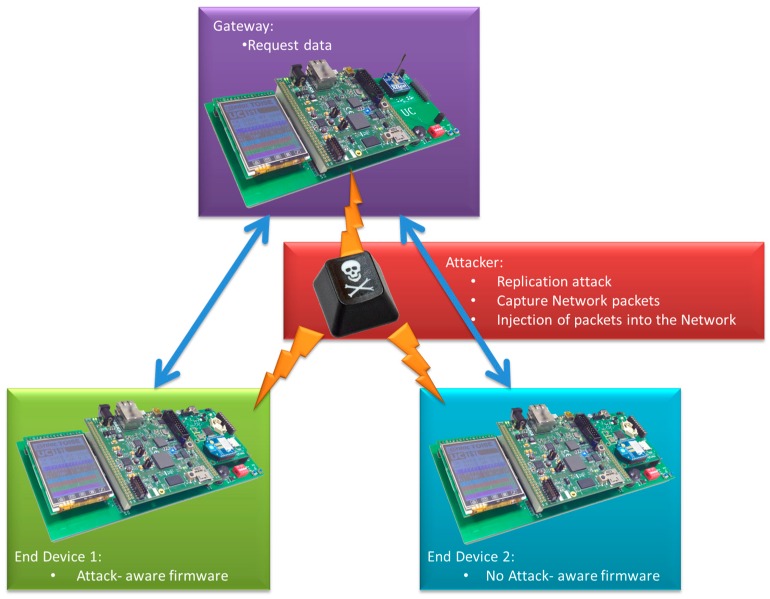
Network topology for accuracy evaluation.

In order to evaluate the proposed methodology, an example of attacked WSNs has been simulated. The WSN includes three nodes (see Figure 12). One of them is a gateway that communicates the WSN with external networks using an additional modem (a GPRS in the proposed example). All the nodes have different hardware architecture and different embedded software. Node 1 has an attack-aware firmware that was designed after using the proposed methodology. The aim of this firmware is to reduce the replication attack effects.

The HW platform of the nodes has a STM32F4 device with an ARM processor, a memory, a temperature sensor, a passive infrared sensor (PIR), a humidity sensor and an 802.15.4 transceiver. All components are interconnected by a system bus. The difference between the HW architecture of node 1 and node 2 is that node 1 includes a HW crypto and node 2 uses a software crypto library. The gateway includes an additional GPRS modem. The gateway’s embedded application software requests information from the end devices every 3 s. The end devices are coordinated and wait to receive the request from the gateway. When they receive the gateway request, they read the sensors, encrypt the information and send it to the gateway. After this, they enter in the sleep mode for 3 s.

With the use of the described methodology, an attack-aware firmware is implemented in node 1. The process used to design this firmware is described below. In the first stage, a battery of attacks was introduced in the simulator to characterize the impact of every attack type and identify the most dangerous attacks. The comparison between the normal network operation and the behavior under attack identifies the specific effect of an attack. These effects allow classifying the attacks in terms of potential hazard. In this use case, the battery of attacks includes jamming, interrogation, collision, hello flood and replication attack with different configuration. The attack analysis identifies the most dangerous attack (in this case, the replication attack) that produces the higher power consumption increase. The hazard of this attack is a consequence of the node behavior. The end-device application is developed to enter in a low power mode (sleep mode) when it does not receive requests from the gateway. During a replication attack, the end-device continually receives request packets, and it never enters the sleep mode, increasing the node consumption drastically. Others attacks shows a low increment in the power consumption of the nodes that reduce their hazard.

Once vulnerability in the network is detected, the second stage is applied. The parameters that are affected by the attack (in this case the rate of the received packets in the end device nodes) are estimated with a non-attack simulation and their values are compared with the simulation under attack. The analysis of the replication attack impact shows that an end-device node without attacks receives a packet every 3 s or 0.33 packets/s. If the network is simulated with a replication attack, the packet rate will increase to more than 0.33 packets/s. In order to detect the attack, a maximum received packet rate could be defined. In this case the selected rate is 1 packet every 3 s (0.33 packets/s). If the attack introduces a higher rate it will be detected and the countermeasure will be executed.

**Figure 13:**
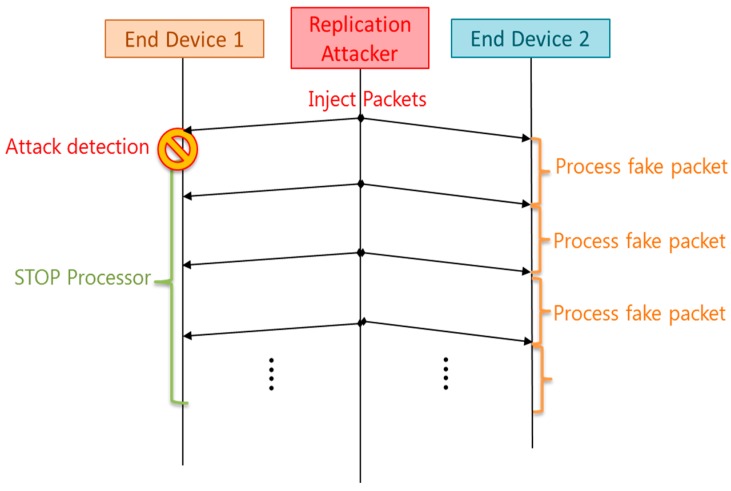
Firmware running under replication attack conditions.

Once the attack is detected, a countermeasure has to be implemented. Different attack-aware methods were introduced and compared in the simulation. It was observed that the most effective response against the attack was to sleep the node once the receiving packet rate was higher than the defined threshold rate. This sleep mode was configured to wait for a specific amount of time (30 s). After that, it awakes and continues searching for the replication attack. The idea is to avoid the draining of the battery with this firmware response. A scheme of this countermeasure is shown in Figure 13, where it can be appreciated that the end device 1 detects the attack and stops its processor. However, the end device 2 does not have countermeasures and it processes all the fake packets injected by the attacker, thus increasing its power consumption.

In order to validate this methodology, the network in Figure 12 was physically implemented and its real power consumptions were measured and compared with the simulator estimations. The real power consumption and execution time has been measured with an Agilent N6705b DC Power Analyzer [72]. To implement the physical attack, an additional component in the network is included: an attacker. This attacker implements a replication attack. It consists in repeatedly sending packets to the destination node. These packets were previously captured on-air by a RZUSBSTICK [73] so they can be correctly identified by the victim. After that, the RZUSBSTICK injects continuously the captured packets in the network. The idea behind this attack is to diminish the device battery life by forcing the node to process useless packets. The attack rate is defined as 1 packet every 2 s, thus it will be detected by the attack-aware firmware.

The following tables compare results between node 1 that has the attack-aware firmware and the node 2 that has a firmware with no countermeasures against attacks.

Table 7 shows the different real power consumptions of the network for different conditions. The time slot was 2 min. It can be observed that in the case of an attack, the power consumption increases 132% compared to the attack-aware firmware. Moreover, the consumption of the two nodes in a normal operation (without attacks) is similar. This proves that the countermeasures developed do not increase the consumption in normal operation.

#### Results

The embedded software of the end devices was evaluated in the proposed virtual platform and in a real environment. The impact of the replication attack was also analyzed in the second experiment. Table 8 shows the power consumption of the end devices after 2 min of execution. During this period, the end devices were under attack. It can be observed that the node that has an attack-aware firmware (end device 1) requires less than 50% of the power of the other node. The virtual platform results show a similar behavior. It is important to remark that the Table 8 simulation results are compared with the real physical measurement.

Additionally, the virtual platform estimations are quite accurate. In this example, the estimation error of the virtual platform is only 8%. In terms of power consumption and execution time, the accuracy of the results is similar to other native-simulation based approaches [54]. Thus, the proposed virtual platform can be used to evaluate the WSN network behavior even when the WSN is not deployed and it is not possible to perform real measurements.

## 7. Conclusions

During the last years, there has been an increasing interest in WSN security. WSN attacks not only disrupt WSN behavior and allow access to restricted data but also increase the power consumption of the attacked nodes and reduce their battery life. The improvement in WSN security is a challenging problem because there is a wide variety of possible attack strategies that have to be analyzed.

This paper proposes a methodology to increase WSN security that includes three main contributions. Firstly, a methodology that allows the development of attack-aware embedded software. Secondly, an attack model that integrates some of the most important WSN attacks with only four categories. This model is used in the last contribution: a virtual platform that can simulate WSN under attacks.

The attack model that has been presented in this paper is focused on active attacks, which mostly affect the network performance. More precisely, it focuses on those attacks that try to disrupt, totally or partially, the communication flow among network nodes. Three types of attacker nodes have been identified: Link-noise, Fake-packet injection and Direct attack nodes. These attackers cover most of the vulnerabilities for WSN.

The proposed virtual platform is able to model the node’s HW, RTOS, embedded SW, and wireless network. Additionally, the tool also models attacks over the WSN. All WSN attacks are modeled by using the attackers specified in the previously-defined attack model. Thus, the simulator can estimate the impact on behavior or power consumption that any node or the whole WSN network suffers under an attack.

Moreover, a methodology is proposed to design a secure Wireless Sensor Network. With this methodology it is possible to design secure firmware that prevents WSN attacks. The proposed technique enables the detection of the most problematic attacks for specific networks. Moreover, it allows the test and selection of the most effective countermeasure firmware before the deployment of the network.

Experimental results show the impact of specific attacks on different types of networks. Additional experiments demonstrate that the estimation error (about 8%) is good enough to enable the simulator to be used for early performance analysis.

## Figures and Tables

**Table 1:** Survey of simulators.

Simulator	Traffic Generation	Real SW Code Support	HW Platform	OS Support	Power Consumption	Security Measure	Limitations
NS-2 (The Network Simulator)	Traffic patterns	NO	NO	NO	YES	NO	No real traffic
NS-3	Traffic patterns	NO	NO	NO	YES	NO	No real traffic
TOSSIM	Statically or Dynamically	Only TinyOS	NO	TinyOS	With PowerTOSSIM	NO	Only for TinyOS code
UWSim	Dynamically	NO	YES	NO	NO	NO	Only for Under Water networks
Avrora	Real	YES	Limited	NO	YES	NO	Only for Mica2 sensor nodes
Castalia	Real	YES	NO	NO	YES	NO	Not a sensor specific platform.
GloMoSim	Statistical	NO	NO	NO	NO	NO	Statistical traffic, no energy models
Shawn	Not real	NO	NO	NO	NO	NO	No real traffic
J-Sim	Not real	NO	NO	NO	YES	NO	Low efficiency. No real traffic
Prowler	Probabilistic	NO	MICA (AVR) Mote	TinyOS	NO	NO	Probabilistic traffic.
ATEMU	Real	YES	AVR processor based systems	TinyOS	YES	NO	Only for AVR processor based systems
OMNeT++	Events	NO	YES With extension	NO	YES	NO	Slow. No real SW code
COOJA	Real	YES	YES	Contiki OS	YES	NO	Low efficiency. Limited number of simultaneous node types.
Proposed Simulator	Real	YES	YES	Multiple	YES	YES	

**Table 2:** Relation between proposed attackers and real WSN attacks.

Attack	Main Effect of the Attack	Attacker Used
**Jamming**	Reduction of the network traffic (disrupts network functionality)	Link-Noise attacker
**Collision**	Reduction of the network traffic (packet corruption)	Link-Noise attacker
**Resource Exhaustion**	Reduction of the network traffic	Link-Noise attacker
**Black Hole Attack**	Reduction of the network traffic (attracting all the packets to the attacked node destination, and silently discarding or dropping them)	Link-Noise attacker
**Path-based DoS**	Reduction of the network traffic	Link-Noise attacker
**Homing**	Reduction of the network traffic (disable attacked nodes)	Link-Noise attacker
**Selective Forwarding**	Reduction of the network traffic	Link-Noise attacker
**Interrogation**	Injects “RTS” packets into the network	Fake packet Injector attacker
**Energy Drain**	Injects fabricated reports	Fake packet Injector attacker
**Hello Flood**	Injects “Hello” packets	Fake packet Injector attacker
**Misdirection**	Routes a packet to distant nodes	Fake packet Injector attacker
**Flooding**	Injects new connection requests	Fake packet Injector attacker
**Spoofed**	Modification of the network routing (Removing some packets & injecting new packets)	Link + Injector attacker
**Sybil**	Modification of the network routing (Removing some packets & injecting new packets)	Link + Injector attacker
**Looping in the net**	Modification of the network routing (Removing attacked packets & injecting same packets into other destinations)	Link + Injector attacker
**Node Replication**	Isolate some nodes and inject new fake packets (Removing some packets & injecting new packets)	Link + Injector attacker
**Overwhelm Attack**	Overwhelm sensor nodes	Direct attacker
**Application Attack**	Modification of the SW node	Direct attacker
**Data Tampering**	Information theft (no effects) where an adversary gains full control over the node	Not modeled
**Sniffing**	Information theft (no effects)	Not modeled

**Table 3:** Energy consumption estimations for Linear Network.

	Linear Wireless Sensor Network Energy Consumption									
	Node 0	Node 1	Node 2	Node 3	Node 4	Node 5	Node 6	Node 7	Gateway	Total
**No Attacks**	2.47 J	2.47 J	2.47 J	2.47 J	2.47 J	2.47 J	2.47 J	2.47 J	1.33 J	21.11 J
**Jamming**	0%	0%	0%	0%	0%	0%	0%	55%	89%	12%
**Injection (Hello Flood)**	0%	0%	0%	0%	0%	0%	0%	0%	543%	34%
**Jamming + Injection**	0%	0%	77%	326%	38%	0%	0%	0%	0%	45%

**Table 4:** Energy Consumption estimations for Meshed Network.

	Meshed Wireless Sensor Network Energy Consumption									
	Node 0	Node 1	Node 2	Node 3	Node 4	Node 5	Node 6	Node 7	Gateway	Total
**No Attacks**	1.91 J	1.91 J	1.91 J	1.91 J	1.91 J	1.91 J	1.91 J	1.91 J	9.18 J	24.48 J
**Jamming**	102%	102%	102%	102%	102%	102%	102%	102%	91%	98%
**Injection (Hello Flood)**	0%	0%	0%	0%	0%	0%	0%	0%	28%	11%
**Jamming + Injection**	0%	0%	0%	409%	0%	0%	0%	0%	0%	34%

**Table 5:** Energy Consumption estimations for Circular Network.

	Circular Wireless Sensor Network Energy Consumption									
	Node 0	Node 1	Node 2	Node 3	Node 4	Node 5	Node 6	Node 7	Gateway	Total
**No Attacks**	2.66 J	2.66 J	2.66 J	2.66 J	2.66 J	2.66 J	2.66 J	2.66 J	2.93 J	24.21 J
**Jamming**	0%	0%	0%	0%	0%	0%	0%	0%	86%	10%
**Injection (Hello Flood)**	0%	0%	0%	0%	0%	0%	0%	0%	95%	11%
**Jamming + Injection**	0%	0%	41%	189%	0%	43%	0%	0%	0%	30%

**Table 6:** Execution time of the simulator.

	Linear	Meshed	Circular
not attacked	43 s	37 s	45 s
Jamming	40 s	35 s	44 s
Injection	52 s	44 s	55 s
Jamming + Injection	51 s	42 s	53 s

**Table 7:** Energy Consumption results.

Attack	Node 1: Hardware Crypto + Attack Aware Firmware	Node 2: Software Crypto + Insecure Firmware
**Not Attacked**	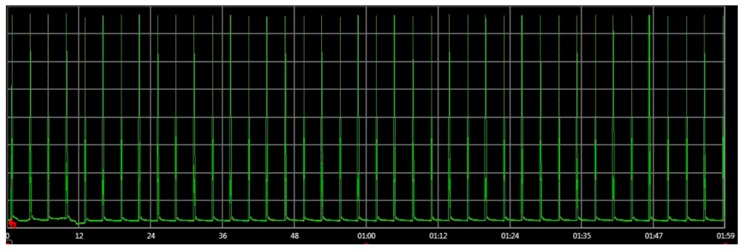	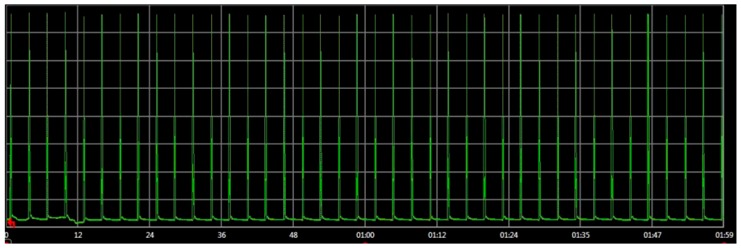
	2.02 mWh	2.02 mWh
**Replication Attack**	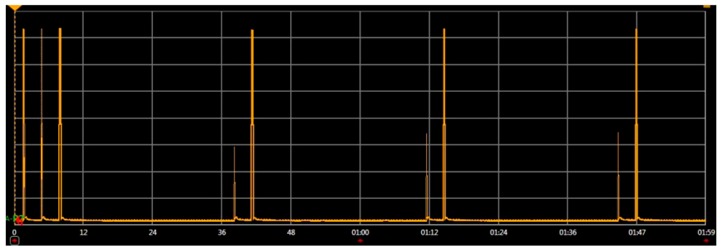	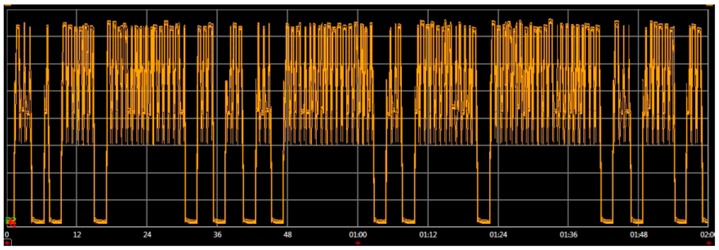
	1.93 mWh	4.48 mWh

**Table 8:** Real/Simulation Consumption Results for WSN.

	WSN under Attack		WSN without Attack	
	Node 1: Attack Aware Firmware	Node 2: Unsecure Firmware	Node 1: Attack Aware Firmware	Node 2: Unsecure Firmware
**Real Measurements**	1.93 mWh	4.48 mWh	2.02 mWh	2.02 mWh
**Virtual Platform Estimations**	2.01 mWh	4.27 mWh	2.17 mWh	2.17 mWh
